# Intrauterine Administration of PBMC Modulated with IFN-τ Before Embryo Transfer Improves Clinical Outcomes of IVF Patients—A Randomized Control Trial

**DOI:** 10.3390/biomedicines14010061

**Published:** 2025-12-26

**Authors:** Margarita Ruseva, Dimitar Parvanov, Rumiana Ganeva, Maria Handzhiyska, Jinahn Safir, Stefka Nikolova, Teodora Tihomirova, Dimitar Metodiev, Georgi Stamenov, Savina Hadjidekova

**Affiliations:** 1 Research Department, Nadezhda Women’s Health Hospital, 1373 Sofia, Bulgaria; dimparvanov@abv.bg (D.P.); rum.ganeva@gmail.com (R.G.); mariavh@abv.bg (M.H.); jinahn.safir@gmail.com (J.S.); 2 Embryology Department, Nadezhda Women’s Health Hospital, 1373 Sofia, Bulgaria; stefka.v.nikolova@gmail.com; 3 Obstetrics & Gynecology Department, Nadezhda Women’s Health Hospital, 1373 Sofia, Bulgaria; t.tihomirova@mail.bg (T.T.);; 4 Pathology Department, Nadezhda Women’s Health Hospital, 1373 Sofia, Bulgaria; 5 Genetics Department, Nadezhda Women’s Health Hospital, 1373 Sofia, Bulgaria; svhadjidekova@medfac.mu-sofia.bg; 6 Department of Medical Genetics, Faculty of Medicine, Medical University-Sofia, 1431 Sofia, Bulgaria

**Keywords:** assisted reproduction, PBMC, IFN-τ, embryo transfer, IVF outcomes

## Abstract

**Objective:** The aim of this study was to evaluate whether intrauterine administration of autologous peripheral blood mononuclear cells (PBMCs) activated with interferon tau (IFN-τ) before embryo transfer improves implantation and pregnancy outcomes in IVF patients. **Methods:** This single-center, prospective, randomized, controlled trial was conducted at Nadezhda Women’s Health Hospital (Approval No.: 6/28022023). The study was registered at ClinicalTrials.gov (NCT05775198). Randomization was computer-generated with allocation concealed via sealed envelopes. Participants and statisticians were blinded to group assignment; clinicians were not. Women aged 21–50 undergoing frozen–thawed embryo transfer with euploid embryos were included. Exclusion criteria included uterine anomalies, autoimmune, oncologic conditions, infections, or use of immunosuppressants. Participants (*n* = 340) were randomized 1:1 to receive either intrauterine infusion of autologous PBMCs activated in vitro with IFN-τ or standard IVF care without PBMC treatment. PBMCs were cultured with recombinant IFN-τ, washed, and infused 24 h prior to single euploid blastocyst transfer. A total of 14 patients were excluded from analysis because of early dropout, leaving 326 (*n* = 167; *n* = 159) patients for modified intention-to-treat analysis. Primary outcomes included implantation rate (elevated urinary or blood hCG), clinical pregnancy (fetal heartbeat at 6–8 weeks), and live birth rates. Miscarriage rate and safety were secondary objectives. Patients were followed up until 6 weeks post pregnancy resolution. **Results:** In the intervention group, 38.3% of patients achieved implantation, compared to 27.7% in the controls (OR 1.6, 95% CI: 1.0–2.6, *p* = 0.04). Live birth rates were also significantly higher in the IFN-τ-modulated PBMC group (28.7% vs. 17.6%, OR 1.9, 95% CI: 1.1–3.2; *p* = 0.02). While the clinical pregnancy rate was higher, it did not reach statistical significance (34.7% vs. 25.8%, *p* = 0.08). There was no difference between the groups in terms of miscarriage (*p* = 0.4). No serious adverse events were reported after treatment, during pregnancy or in the postnatal period. **Conclusions:** Intrauterine treatment with IFN-τ-activated PBMCs before ET significantly improves implantation and live birth rates in IVF patients.

## 1. Introduction

Embryo implantation remains one of the most critical and rate-limiting steps in assisted reproductive technology (ART), with implantation failure accounting for a substantial proportion of unsuccessful in vitro fertilization (IVF) cycles despite transfer of morphologically high-quality or genetically screened embryos [1]. While advancements in embryo culture, cryopreservation, and preimplantation genetic testing have improved ART outcomes, recurrent implantation failure (RIF) continues to affect up to 15% of IVF patients [2].

The successful establishment of pregnancy depends not only on embryo quality but also on precise synchronization between the embryo and a receptive endometrium, which is modulated by a complex interplay of hormonal, cellular, and immune factors [3]. Among these, the maternal immune system plays a pivotal role—not only through not rejecting the semi-allogeneic embryo, but also in actively facilitating its implantation through immune tolerance, cytokine production, and controlled local inflammation [4,5].

In this context, peripheral blood mononuclear cells (PBMCs)—a heterogeneous population comprising varying proportions of T cells, B cells, natural killer (NK) cells, monocytes, and dendritic cells—have emerged as a source of potential modulators of endometrial receptivity and embryo–maternal immune crosstalk [6,7]. In preclinical studies, intrauterine administration of PBMCs—particularly when activated in vitro with human chorionic gonadotropin (hCG)—has been shown to improve markers of endometrial receptivity in animal models. These improvements include enhanced expression of genes and proteins involved in hormone signaling, angiogenesis, and inflammation [8,9]. Building on these findings, intrauterine infusion of autologous PBMCs activated with hCG has been explored as an adjunctive therapy in assisted reproductive technology (ART), particularly for patients with repeated implantation failure. PBMCs are thought to contribute to implantation by secreting cytokines such as IL-6, IL-10, TNF-α, and GM-CSF, which help recruit other immune cells and support key processes like trophoblast invasion, decidualization, and vascular remodeling at the implantation site [10]. The modulator hCG serves as an embryonic signal that induces a tolerogenic shift in PBMCs, enhancing their secretion of anti-inflammatory cytokines and further promoting a uterine environment conducive to implantation [11]. Clinical studies, including randomized controlled trials and meta-analyses, have demonstrated that hCG-modulated PBMCs administered before embryo transfer can significantly increase implantation, clinical pregnancy, and live birth rates, without increasing the risk of miscarriage [10,12,13,14,15].

Despite these promising findings, alternative modulators of PBMC activity may offer improved efficacy or novel mechanisms of action. One such trophoblast-derived compound is interferon tau (IFN-τ), a type I interferon that serves as the maternal recognition signal of pregnancy in ruminants [16]. Although IFN-τ has not been identified in humans, its receptor (IFNAR) is widely expressed on human immune cells, suggesting a potential cross-species mechanism of action [17]. IFN-τ has been shown to exert potent immunomodulatory, anti-viral, anti-apoptotic, and anti-inflammatory effects [16,17,18,19]. Compared to its human analogue IFN-α, IFN-τ has lower cytotoxicity and a more favorable immunological profile, which has led to its investigation in autoimmune, metabolic, and inflammatory disease models, including multiple sclerosis and viral infections [17,18,20].

This randomized controlled trial was designed to evaluate the efficacy and safety of intrauterine administration of in vitro IFN-τ-modulated autologous PBMCs in improving IVF outcomes. We hypothesize that IFN-τ-primed PBMCs will induce a more favorable uterine immune environment, enhancing endometrial receptivity and supporting successful embryo implantation without compromising pregnancy safety.

## 2. Materials and Methods

### 2.1. Study Design, Randomization, and Study Groups

**Figure 1:**
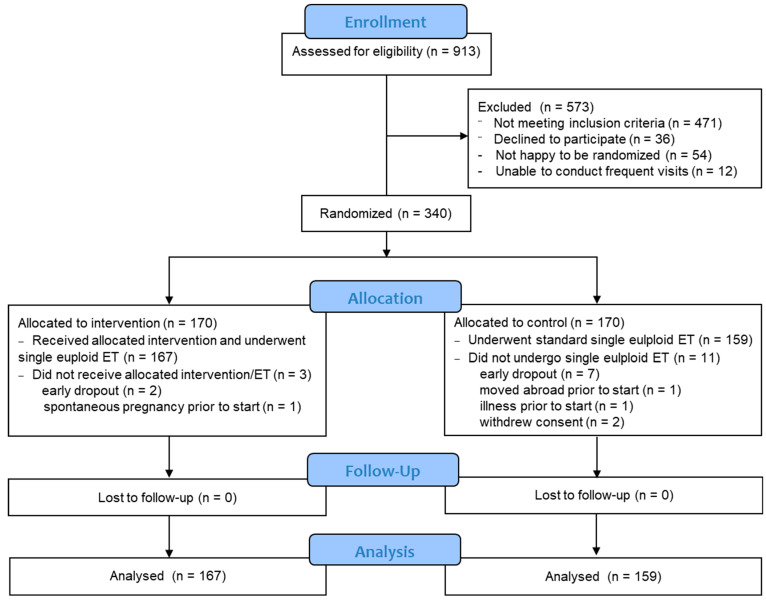
Participant flow through the study. CONSORT flow diagram showing enrollment, randomization, allocation, follow-up, and analysis of participants in the randomized controlled trial evaluating the effect of intrauterine infusion of IFN-τ-activated autologous PBMCs prior to embryo transfer (ET) in IVF patients. Of 913 women assessed for eligibility, 340 were randomized (1:1) to receive either IFN-τ-activated PBMC treatment or standard IVF care. After exclusion of early dropouts, who did not undergo ET, 167 participants in the intervention group and 159 in the control group completed the protocol and were included in the modified intention-to-treat analysis. No participants were lost to follow-up.

A single-center, prospective, randomized, controlled, clinical trial was conducted at Nadezhda Women’s Health Hospital after approval by the Institutional Ethics Committee (Approval No.: 6/28022023, 28 February 2023). The study was prospectively registered at ClinicalTrials.gov under identifier NCT05775198. Participants were randomized (1:1) into the intervention group to receive intrauterine infusion of autologous PBMCs activated in vitro with IFN-τ, and the control group, where patients would undergo a standard IVF protocol and single euploid embryo transfer (ET) without PBMC treatment (Figure 1).

### 2.2. Participants

Eligible participants were female patients undergoing assisted reproduction treatment for primary idiopathic infertility, defined as the inability to achieve a clinical pregnancy without an identifiable cause, who had at least one euploid embryo available for transfer confirmed by preimplantation genetic testing for aneuploidy (PGT-A). All participants had regular menstrual cycles and normal uterine anatomy confirmed by ultrasonography or hysteroscopy. Exclusion criteria included the presence of uterine pathologies (such as polyps, intrauterine adhesions, or fibroids distorting the endometrial cavity), endometrial bacterial infections, active endometrial inflammation, or recent use of immunosuppressive therapy. Patients were also excluded if they had polycystic ovary syndrome, detectable autoantibodies (anti-thyroid peroxidase, anti-thyroglobulin, anticentromere antibodies, antiphospholipid antibodies, antinuclear antibodies, or anti-double-stranded DNA), inherited coagulation disorders (deficiencies in factor XII, protein C, or protein S); any active autoimmune or oncological condition; or seropositivity for HIV, hepatitis B, or hepatitis C.

### 2.3. PBMC Isolation and in Vitro Modulation

Peripheral blood (9 mL) was collected in acid citrate dextrose tubes on stimulation day 3 after progesterone supplementation during endometrial preparation in frozen cycles. Blood was diluted 1:1 with phosphate-buffered saline (PBS) and carefully layered over Pancoll (P04-60100, Pan Biotech, Aidenbach, Germany) (diluted blood/Pancoll 3:1). PBMCs were isolated using density gradient centrifugation at 400× *g* for 30 min at room temperature without brake. After washing with PBS, cells at concentration of 1 × 10^7^ cells/mL were cultured in a humidified incubator for 24 h at 37 °C and 5% CO_2_ in 0.5 mL RPMI-1640 medium (P04-22100, Pan Biotech, Aidenbach, Germany) supplemented with 1 mg/mL human serum albumin (HSA; A1A029AA, Takeda, Zurich, Switzerland), antibiotics mix (penicillin/streptomycin/amphotericin B; P06-07300, Pan Biotech), and recombinant IFN-τ (CSB-YP350007SH, CusaBio, Houston, TX, USA) at a concentration of 500IU.

### 2.4. Intrauterine Administration of Modulated Cells

After 24 h culture, the modulated PBMCs were washed in PBS again, suspended in 0.5 mL sterile saline, and introduced into the uterine cavity using a soft embryo transfer catheter by a trained assisted reproduction specialist. Patients were monitored for 2 h after the procedure and instructed to report any adverse events.

Cell viability (≥90%) and concentration were confirmed prior to intrauterine infusion. Viability was assessed using propidium iodide (PI, P4864-10ML; Sigma-Aldrich, St. Louis, MO, USA) and 7-aminoactinomycin D (7-AAD, 130-111-568; Miltenyi Biotec, Bergisch Gladbach, Germany) staining. After 24 h of incubation under experimental conditions and washing with PBS, a portion of the cells (1 × 10^6^) were stained with PI and 7-AAD according to the manufacturer’s instructions and analyzed by flow cytometry. Viable cells were identified as those negative for both PI and 7-AAD.

### 2.5. IVF Protocol and Embryo Transfer

Patients in both arms underwent standard frozen–thawed embryo transfer. Embryo quality was assessed according to the Gardner morphology grading system, assigning separate scores to the inner cell mass (ICM) and trophectoderm (TE) [21], and PGT-A was performed on day 5 embryos. Only single good-quality euploid blastocysts (grade A or B for both ICM and TE) were transferred. Luteal phase support in the form of vaginal progesterone was provided equally in both groups.

### 2.6. Outcome Measures

Primary outcomes included implantation rate, defined as a positive urinary or serum hCG test 14 days after ET; clinical pregnancy rate (CPR), defined as the presence of a fetal heartbeat at 6–8 weeks of gestation confirmed by transvaginal ultrasound; and live birth rate (LBR).

Secondary outcomes included miscarriage rate and incidence of adverse events. Safety outcomes were recorded throughout the participants’ involvement in the trial and encompassed any adverse medical event in the mother or baby, including neonatal death or congenital abnormalities, defined as abnormalities listed on the European Surveillance of Congenital Anomalies (EUROCAT) Guide 1.5 [22], up to six weeks after birth. All events were evaluated for expectedness and categorized as either expected adverse events (AEs) or expected serious adverse events (SAEs). Expected events were those commonly associated with IVF treatment and intrauterine infusions (Table A1). Any event not included on this list was defined as an unexpected adverse outcome.

### 2.7. Statistical Analysis

To ensure an adequate sample size to detect differences in clinical outcomes, a priori power analysis was conducted to determine the required number of participants in each group. Calculations were performed in Python (v.3.11) using the statsmodels package (v. 0.13.2), assuming a 30% implantation rate, 25% CPR, and 20% LBR in the control group and a 10% absolute increase following PBMC treatment. With a two-sided alpha level of 0.05 and 80% power, the analysis indicated that a minimum of approximately 160 participants per group was required. To confirm that the study retained adequate power for the primary endpoints, a post hoc power analysis was performed using the observed event rates for implantation, clinical pregnancy, and live birth. Power was calculated for two-proportion comparisons based on the final analyzed sample.

Outcomes were analyzed in the per-protocol population, defined as all participants who underwent embryo transfer and had outcome data available. Participants who withdrew early and no embryo transfer was performed were excluded from the primary analysis. To assess the robustness of the findings and ensure the treatment effect was not overestimated due to attrition bias, a sensitivity analysis addressing missing outcome data was conducted. Specifically, a conservative approach was adopted, assuming that all participants with missing outcomes did not achieve the primary endpoints.

All data were analyzed using SPSS version 27.0 (IBM Corp., Armonk, NY, USA) and visualizations were performed using Python (v. 3.11). Normality was assessed using the Shapiro–Wilk test. Comparisons between study groups were performed via Chi-square or Fisher’s exact tests for categorical variables (outcomes) and the independent samples Mann–Whitney U test for continuous variables (baseline characteristics). Data are presented as mean ± standard deviation (SD) or median [IQR], as appropriate. A *p*-value < 0.05 was considered statistically significant.

## 3. Results

### 3.1. Patient Flow and Baseline Characteristics

A total of 340 participants were enrolled and randomized equally between the intervention (*n* = 170) and control (*n* = 170) groups (Figure 1). Baseline demographics and clinical characteristics were comparable between the two groups, ensuring homogeneity across the cohorts (Table 1).

### 3.2. Primary Outcomes

The primary outcomes were analyzed in the per-protocol population, defined as all participants who underwent embryo transfer and had outcome data available. Participants who withdrew early or had no euploid embryos available for transfer (3 in the treatment group and 11 in the control group) were excluded from the primary analysis (Figure 1). A true intention-to-treat analysis was not feasible due to missing outcome data.

The implantation rate, defined by a positive serum β-hCG, was significantly higher in the group receiving intrauterine infusion of IFN-τ-activated PBMCs compared to the control group (38.3% vs. 27.7%, odds ratio (OR) 1.6, 95% confidence interval (CI): 1.0–2.6; *p* = 0.04).

Although the clinical pregnancy rate, defined by the presence of a fetal heartbeat at 6–8 weeks of gestation, was higher in the intervention group (34.7%) compared to the controls (25.8%), this difference did not reach statistical significance (*p* = 0.08).

The live birth rate was significantly improved in the intervention group, with 28.7% of women achieving live births, compared to 17.6% in the control group (OR 1.9, 95% CI: 1.1–3.2; *p* = 0.02).

Using the observed implantation rates, the post hoc power was approximately 79%. For the live birth outcome, the post hoc power was approximately 84%. The observed effect size for clinical pregnancy yielded a lower post hoc power of approximately 63%, consistent with the nonsignificant *p*-value for this endpoint. These analyses confirm that the study retained adequate power for two of the primary outcomes, while the clinical pregnancy outcome was comparatively underpowered.

**Figure 2:**
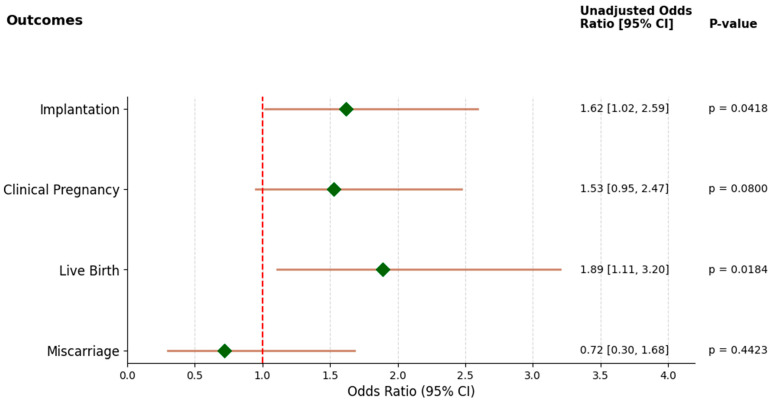
Treatment effect of IFNτ-activated PBMC on IVF outcomes. Forest plot showing unadjusted odds ratios (ORs) with 95% confidence intervals (CIs) for implantation, clinical pregnancy, live birth, and miscarriage. Green diamonds indicate the point estimate of the OR for each outcome, horizontal brown lines represent 95% CIs, and the vertical red dashed line denotes the null value (OR = 1.0), indicating no difference between groups. ORs to the right of 1.0 indicate increased odds of the outcome in the treatment vs. control group, while ORs to the left of 1.0 indicate decreased odds of the outcome in the treatment vs. control group. Outcomes whose 95% CI does not cross OR = 1.0 are considered statistically significant, corresponding to *p* < 0.05. The ORs and corresponding 95% CIs are shown numerically to the right, alongside their *p*-values.

The forest plot (Figure 2) reflects the per-protocol population. Participants without embryo transfer or outcome data (*n* = 14) were excluded from the analysis.

Implantation and live birth outcomes show statistically significantly increased odds, while the observed changes in clinical pregnancy and miscarriage rates are not statistically significant.

### 3.3. Secondary Outcomes

Miscarriage rates did not differ significantly between the two groups (*p* = 0.4). No unexpected or serious expected adverse events or complications related to the PBMC infusion or embryo transfer were reported, suggesting the procedure is safe and well-tolerated. The incidence of expected mild to moderate adverse events was similar between the two groups (Table 2). Those were limited to mild pelvic pain and cramping, fatigue, abdominal discomfort and bloating, and vaginal spotting. There was no incidence of intrauterine death, fetal growth restriction, or congenital anomaly detected at birth or in the early postnatal period in either of the study groups.

A sensitivity analysis assuming all excluded participants did not achieve the studied outcomes showed preserved direction and magnitude of treatment effect across all outcomes, supporting robustness of the per-protocol findings (Table A2).

## 4. Discussion

This randomized controlled trial demonstrates that intrauterine administration of IFN-τ-activated autologous PBMCs prior to frozen–thawed euploid blastocyst transfer significantly enhances implantation and live birth rates in IVF patients, without increasing miscarriage risk or adverse maternal outcomes. These findings suggest a promising immunomodulatory strategy for improving endometrial receptivity and embryo implantation success, particularly in the context of single euploid embryo transfer protocols.

While the analysis was performed per embryo transfer rather than intention-to-treat, the dropout rate was low for an IVF study (1.8% in the intervention group and 6.5% in controls). The observed 10.6% absolute increase in implantation rate and 11.1% increase in live birth rate are clinically meaningful, especially given the growing use of frozen–thawed transfers (Table 2). Control group outcomes were consistent with published benchmarks for women of comparable maternal age [23], supporting the internal validity of these results. In the current study, although the clinical pregnancy rate was higher in the IFN-τ-PBMC group, the difference did not reach statistical significance, likely due to natural variability in early pregnancy loss. Importantly, miscarriage rates were not elevated, indicating that the observed improvements in implantation did not compromise early pregnancy stability.

A notable strength of this study is its prospective, randomized design and the exclusive use of PGT-A—confirmed euploid embryos—which minimizes confounding related to embryo quality and ensures that the observed differences are more likely attributable to the intervention. Additional strengths include clearly defined eligibility criteria and standardized procedures for PBMC preparation and intrauterine administration, supporting internal validity. Nonetheless, several limitations warrant acknowledgment. The single-center design may limit generalizability, and the lack of clinician blinding and absence of a sham intrauterine procedure in the control group may introduce the potential for performance bias. Nonetheless, these design choices were driven by ethical considerations and, importantly, the primary outcomes of biochemical pregnancy, clinical pregnancy, and live birth were objectively measured, which mitigates the likelihood of significant bias. Finally, while early dropout was minimal and unlikely to meaningfully influence the findings, multicenter studies will be important to confirm the validity and broader applicability of these results. The rationale for using IFN-τ as a priming agent for PBMCs stems from its unique immunological profile. Originally identified in ruminants as a pregnancy recognition signal, IFN-τ promotes maternal immune tolerance and endometrial receptivity by altering cytokine expression, promoting leukocyte recruitment, and stimulating interferon-stimulated genes (ISGs) [17,24]. Preclinical mechanistic evidence from bovine endometrial models suggests that IFN-τ protects epithelial cells from inflammation-induced damage and promotes M2 polarization in the endometrium [25,26]. While IFN-τ is not endogenously produced in humans, its receptor (IFNAR) is expressed on human immune and endometrial cells, allowing for cross-species bioactivity [27,28].

Our findings are consistent with preclinical evidence demonstrating that IFN-τ has potent immunomodulatory and anti-inflammatory effects beyond its original reproductive context. In bovine models, IFN-τ is secreted as early as day 7 by the conceptus, inducing an anti-inflammatory immune response in PBMCs, including upregulation of IL-10 and suppression of Th1 cytokines (TNFα, IL-1β) [29]. Additionally, peripheral blood leukocytes in ruminants exposed to IFN-τ upregulate ISGs such as MX2 and ISG15, correlating with systemic pregnancy markers [30]. These studies support the concept that immune cells primed with IFN-τ can respond in a pregnancy-supportive manner, potentially improving endometrial receptivity.

The effects of IFN-τ are not limited to ruminant species or reproductive tissues. In murine models of immune-mediated pregnancy loss, exogenous IFN-τ prevents fetal resorption in an IL-10-dependent manner [31]. In autoimmune disease models such as experimental allergic encephalomyelitis and type 1 diabetes (diabetic mice and rats), IFN-τ administration via various routes induces a Th2 immune shift, delays disease onset, and reduces inflammatory and oxidative stress damage [32,33,34,35]. Mechanistic insight from one such study suggests IFN-τ reduces proinflammatory cytokines (IL-6, TNF-α) and M1 macrophages, while increasing anti-inflammatory M2 macrophages [36]. This underscores its broader immunological utility and reinforces its role as a tolerogenic agent capable of modulating systemic immune responses.

Importantly, human cells also respond directly to IFN-τ. Tanikawa et al. demonstrated that human trophoblast cell lines treated with IFN-τ show increased expression of cytokines such as IL-6 and IL-8, and enhanced cell proliferation, migration, and mitochondrial antioxidant activity [37]. These effects were mediated via STAT3 activation, supporting the notion that IFN-τ may promote trophoblast–endometrial dialogue, a critical component of successful implantation [37]. Recently, our group demonstrated that IFN-τ promotes a Th2-biased immune shift in PBMCs from RIF patients in vitro by decreasing proinflammatory cytokine (TNFα and IL-6) secretion and improving T cell subset balance, as reflected by increased T helper/cytotoxic T cell ratios and reduced Th1/Th2 and Th17/Treg ratios. Moreover, we observed a significant increase in T helper cell abundance in paired endometrial biopsies following intrauterine administration of IFN-τ-modulated PBMCs [38]. These findings confirm that IFN-τ is biologically active in human immune cells despite its evolutionary specificity to ruminants, and highlight its potential as an immunomodulatory agent in reproductive medicine.

Collectively, these results contribute to a growing body of literature suggesting that periconceptional immunomodulation, particularly via autologous immune cell therapies, can enhance ART outcomes. The use of IFN-τ to activate PBMCs prior to intrauterine administration offers a novel strategy to enhance their local paracrine signaling and skew the uterine immune environment toward a tolerogenic, implantation-supportive state. Future studies incorporating molecular pathway activation and gene expression changes in PBMC and endometrial samples post treatment could help elucidate the specific mechanisms involved in the observed improved reproductive outcomes. Additionally, long-term follow-up studies are warranted to confirm the safety and durability of outcomes associated with IFN-τ-PBMC therapy.

## 5. Conclusions

Intrauterine infusion of autologous PBMCs activated with IFN-τ in vitro represents a safe, well-tolerated, and clinically beneficial adjunctive strategy in IVF. By harnessing IFN-τ’s potent anti-inflammatory and immune-regulating properties, this approach may improve implantation and live birth rates without compromising early pregnancy stability. These findings open promising avenues for immunologically informed fertility treatments and justify further investigation in larger, multicenter clinical trials.

## Figures and Tables

**Table 1:** Baseline characteristics and reproductive history of the study cohort.

Characteristic	Intervention Group (*n* = 170)	Control Group (*n* = 170)	*p*-Value
Age at ET, y	38.2 ± 6.4	38.0 ± 5.5	NS
Reproductive history			
Prior unsuccessful IVF attempts			NS
0, *n* (%)	15 (8.8%)	18 (10.6%)	
1, *n* (%)	33 (19.4%)	27 (15.9%)	
2, *n* (%)	47 (27.6%)	45 (26.5%)	
≥3, *n* (%)	75 (44.1%)	80 (47.1%)	
Duration of infertility, y	6.0 (5.0)	5.0 (5.0)	NS
Current attempt characteristics			
Oocytes collected, *n*	8.5 (5.0)	9.0 (5.0)	NS
Blastocysts, *n*	2.8 ± 1.3	2.9 ± 1.4	NS
Euploid embryos, *n*	1.6 ± 0.7	1.7 ± 0.7	NS

Data are presented as mean ± standard deviation (SD) or median (IQR), as appropriate. NS—not significant.

**Table 2:** Absolute difference in study outcomes between the two groups.

Outcome	Intervention Group (*n* = 167)	Control Group (*n* = 159)	Absolute Difference	*p*-Value
Implantation, *n* (%)	64 (38.3%)	44 (27.7%)	+10.6%	0.04
Clinical Pregnancy, *n* (%)	58 (34.7%)	41 (25.8%)	+8.9%	NS
Live Birth, *n* (%)	48 (28.7%)	28 (17.6%)	+11.1%	0.02
Miscarriage, *n* (%)	10 (6.0%)	13 (8.2%)	−2.2%	NS
Incidence of AEs				
Expected				
Mild to moderate, *n* (%)	28 (16.8%)	31 (19.5%)	−2.7%	NS
Severe, *n* (%)	0 (0%)	0 (0%)	0%	NS
Unexpected, n/N	0 (0%)	0 (0%)	0%	NS

NS—not significant.

## Data Availability

The anonymized data supporting the findings presented in this study are available on request from the corresponding author.

## Abbreviations

The following abbreviations are used in this manuscript:

PBMCs	Peripheral Blood Mononuclear Cells
IFN-τ	Interferon tau
IVF	In Vitro Fertilization
ART	Assisted Reproductive Technology
RIF	Recurrent Implantation Failure
NK	Natural Killer
hCG	Human Chorionic Gonadotropin
IL	Interleukin
TNF-α	Tumor Necrosis Factor alpha
GM-CSF	Granulocyte-Macrophage Colony-Stimulating Factor
IFNAR	Interferon Alpha/Beta Receptor
PGT-A	Preimplantation Genetic Testing for Aneuploidy
HSA	Human Serum Albumin
RPMI	Roswell Park Memorial Institute medium
ET	Embryo Transfer
CPR	Clinical Pregnancy Rate
LBR	Live Birth Rate
AEs	Adverse Events
SAEs	Serious Adverse Events
EUROCAT	European Surveillance of Congenital Anomalies
SD	Standard Deviation
IQR	Interquartile Range
OR	Odds Ratio
CI	Confidence Interval
ISGs	Interferon-Stimulated Genes

## Appendix A

The following table contains a list with details on the possible side effects to be expected.

**Table A1:** List of possible expected adverse events associated with IVF, intrauterine infusion, and embryo transfer (ET).

Category and Type of Adverse Event (AE)		Notes
Mild to moderate ^1^		
	Abdominal discomfort or bloating	Common post ovarian stimulation
	Mild pelvic pain or cramping	Possible after egg retrieval, intrauterine infusion, ET
	Breast tenderness	Due to hormonal changes
	Nausea	Common with fertility medications
	Fatigue	Hormonal and stress-related
	Headache	Side effect of gonadotropins or stress
	Mood changes	Usually hormone-related
	Hot flushes	Often related to GnRH agonists or antagonists
	Vaginal spotting or bleeding	Especially post egg retrieval or ET
	Injection site reactions	Pain, redness, or swelling
	Acne	Due to hormone fluctuations
	Constipation	Caused by reduced activity, hormones, and stress
	Sleep disturbances	Stress or medication-related
	Fluid retention	Due to high estrogen levels
	Weight gain	Can be fluid-related or hormonal
	Changes in libido	Hormonal and psychological factors
	Mild allergic reactions	To progesterone or other medications
Serious or significant ^2^		
	Ovarian hyperstimulation syndrome (OHSS)	Complication after ovarian stimulation
	Hospitalization for rest	Due to OHSS or early pregnancy symptoms
	Gestational diabetes	Can occur during IVF pregnancy
	Preeclampsia	Can occur during IVF pregnancy
	Fever	Possible inflammation after treatment

^1^ Mild to moderate AEs—common, generally self-limiting, and usually resolved without medical intervention; ^2^ Serious or significant AEs—requiring close monitoring or medical intervention.

**Table A2:** Sensitivity analysis assuming all missing participants had a negative outcome.

Outcome	Intervention Group (*n* = 170)	Control Group (*n* = 170)	Absolute Difference	*p*-Value
Implantation, *n* (%)	64 (37.6%)	44 (25.9%)	+11.7%	0.04
Clinical Pregnancy, n (%)	58 (34.1%)	41 (24.1%)	+10.0%	NS
Live Birth, *n* (%)	48 (28.2%)	28 (16.5%)	+11.7%	0.02
Miscarriage, *n* (%)	10 (5.9%)	13 (7.6%)	–1.7%	NS

NS—not significant.
